# Effects of Heat and Hypoxia Training on the Fat Oxidation Capacity of Competitive Athletes

**DOI:** 10.1002/ejsc.12312

**Published:** 2025-05-08

**Authors:** Zhizhong Geng, Xiaameng Wu, Jinhao Wang, Guohuan Cao, Chenhao Tan, Longji Li, Jun Qiu

**Affiliations:** ^1^ School of Health Sciences Shanghai University of Sport Shanghai China; ^2^ Loughborough University Leicestershire UK; ^3^ Shanghai Research Institute of Sports Science Shanghai China

**Keywords:** competitive athletes, fat oxidation capacity, heat, hypoxia, training

## Abstract

This study aimed to evaluate the impact of a four‐week heat and hypoxia training on the fat oxidation capacity of competitive athletes. Eight elite male modern pentathlon athletes completed a four‐week aerobic endurance training program in three environments: normal (CON), high temperature and humidity (HOT), and hypoxia (HYP). Assessments were conducted in both the normal environment and the corresponding special environment before and after training. Gas exchange data were collected during exercise to assess aerobic capacity, and fat oxidation was measured using indirect calorimetry. Fat oxidation kinetics were modeled using the sinusoidal (SIN) mathematical model to determine the maximum fat oxidation (MFO) and the exercise intensity at which it occurred (FAT_max_). Under normal environment, HOT training had an increase in absolute V̇O_2_ (238.152 mL/min and *p* = 0.003), both the HOT (96.062 s and *p* = 0.006) and HYP (109.917 s and *p* = 0.002) trainings demonstrated increases in VT_2_@Time, both the HOT (0.126 g/min and *p* = 0.015) and HYP (0.157 g/min and *p* = 0.004) trainings showed increases in MFO, and the HOT training also exhibited an increase in FAT_max_ (5.303 g/min and *p* = 0.005); both the HOT and HYP trainings showed dilatation of the fat oxidation curve, with the HOT training also displaying dilatation in the fat oxidation curve under heat conditions. Four‐weeks of heat and hypoxia training significantly enhanced athletes' aerobic metabolism and fat oxidation capacity. The benefits of heat training on aerobic metabolism and fat oxidation may exceed those of hypoxia training.

**Summary**

- Understanding how extreme environmental conditions, such as heat and hypoxia, influence athletes' metabolic characteristics is crucial for developing tailored training and nutritional strategies to optimize performance and mitigate adverse effects.
- This study evaluates the effects of a four‐week heat, hypoxia, and normal training program on athletes' fat oxidation and aerobic metabolism, revealing distinct responses to environmental factors.
- Heat and hypoxia training improved aerobic capacity and fat oxidation under normal conditions, with heat training yielding superior benefits, whereas hypoxia did not enhance fat oxidation under hypoxic conditions.

## 1 Introduction

Competitive athletes frequently face unique training and competition environments, such as high temperature, high humidity, and low oxygen conditions (Racinais et al. 2015; Rodríguez et al. 2015). These environmental stressors can impair athletic performance, reduce exercise duration, and negatively affect athletes' aerobic metabolic capacity (Geng et al. 2024; Jung et al. 2021; Osawa et al. 2017). In response to these challenges, recent research suggests that adaptive training under specific environmental and exercise intensity parameters in such environments has become an effective strategy for mitigating the negative effects and improving athletic performance (Moss et al. 2020; Nybo et al. 2022). For example, training in high temperature and high humidity conditions has been shown to reduce the body's limitations on cardiac output and skeletal muscle blood flow (Bassett and Howley 2000), thus enhancing performance in hot environments (González‐Alonso et al. 2008). A meta‐analysis further supports that heat acclimation can improve endurance in both hot and normal environments and can moderately increase maximal oxygen uptake (Waldron et al. 2021). Similarly, studies on hypoxic training at moderate altitudes demonstrate performance improvements in endurance athletes (Bonetti and Hopkins 2009; Girard et al. 2023), showing that hypoxic training accelerates erythropoiesis, increases hemoglobin mass, and enhances nonhematological factors, such as mitochondrial efficiency, thereby improving athletes' aerobic metabolic capacity (Gore et al. 2007; Saunders et al. 2013).

Improvement in endurance and aerobic metabolic capacity after training in special environments may also be associated with changes in the body's energy metabolism. Research indicates that the enhancement of athletic performance following endurance training at a given exercise intensity correlates with increased free fatty acid (FFA) oxidation (Achten and Jeukendrup 2003). Fat oxidation capacity may be a critical factor in determining exercise performance (Frandsen et al. 2017), as the body's energy supply during prolonged exercise increasingly relies on fat oxidation (Stisen et al. 2006). This reduces the dependence on carbohydrate oxidation, conserves glycogen stores (Brooks and Mercier 1994), and enhances performance in endurance events (Holloszy and Coyle 1984). Recently, incremental load exercise tests have been widely used to determine whole‐body fat oxidation rates, including maximum fat oxidation (MFO) and the exercise intensity at which MFO occurs (FAT_max_), under various conditions (Achten et al. 2002; Zurbuchen et al. 2020). In addition to these metrics, the sinusoidal (SIN) mathematical model proposed by Chenevière et al. (2009) can accurately predict MFO and provide dynamic parameters—such as expansion, symmetry, and translation—to describe the trends of the fat oxidation curve. Enhanced fat oxidation capacity is often accompanied by an expanded and rightward‐shifted fat oxidation curve (Chenevière et al. 2010, 2011).

Research on special populations, such as patients and individuals with obesity, suggests that training in high temperature, high humidity, or hypoxic environments can lead to beneficial metabolic adaptations. For instance, Mähler et al. (2018) found that intermittent hypoxic training improved FFA oxidation during exercise in patients with multiple sclerosis. Long‐term passive heat exposure has also been shown to reduce endogenous glucose breakdown, shift substrate oxidation toward greater fat utilization, and lower plasma FFA and cholesterol levels (Pallubinsky et al. 2020). Despite the known benefits of environmental training on fat oxidation in certain populations, few studies have explored these effects in competitive athletes. Specifically, there is limited research on how fat oxidation capacity and fat oxidation curve parameters change in athletes under special environmental conditions.

Therefore, this study employed a simulated low‐oxygen environment equivalent to 2500 m altitude and a high temperature (35°C) and high humidity (70% relative humidity) environment for training and testing (Nybo et al. 2022). Using the SIN mathematical model and indirect calorimetry, we compared the effects of 4‐week training in these environments on athletes' fat oxidation capacity in both normal and special environments. The aim was to clarify how different environmental training conditions affect energy metabolism and to explore whether similar improvements in fat oxidation occur under the corresponding environmental stressors. Based on previous studies, we hypothesized that both high temperature and high humidity training and hypoxia training would enhance the aerobic and fat oxidation capacities of competitive athletes, both in normal and the corresponding special environments.

## 2 Methods

### 2.1 Subject

Sample size calculations were performed using the G*Power 3.1 software (Franz Faul, University Kiel, Germany), with a significance level set at *α* = 0.05 and a power of 1 − *β* = 0.90. Based on our previous study (*ηp* ^2^ = 0.191) (Geng et al. 2024), the required effect size was calculated as 0.486, which indicated a minimum sample size of 7 participants. To account for potential participant dropout, we expanded the recruitment and ultimately enrolled 8 male elite modern pentathlon athletes (age: 17.91 ± 2.94 years; height: 1.81 ± 0.06 m; weight: 70.95 ± 8.38 kg; and BMI: 21.69 ± 1.83 kg/m^2^). None of the participants had undergone any training related to hot–humid or hypoxia in the three months preceding the study, and all athletes resided in areas with temperate climates at low altitudes. Modern pentathlon, an Olympic event consisting of five disciplines (fencing, freestyle swimming, equestrian show jumping, pistol shooting, and cross‐country running), places athletes under extreme energy and physiological demands, with competition durations lasting up to 8 h (Le Meur et al. 2012). Given the nature of the sport, modern pentathlon athletes are classified as endurance athletes (Hoffmann et al. 2020). All participants were informed of the study procedures and provided written informed consent prior to the experiment.

### 2.2 Study Design

Eight professional athletes initially underwent a 4‐week training period under normal conditions (control training, CON and *n* = 8). Body composition assessments and an incremental load exercise tests were conducted both before and after this training phase. Given that the training program employed in this study mirrors the athletes' routine training regimen, and considering the continuous nature of professional athletic training, no additional washout period was introduced between the control training and the subsequent special environmental interventions. Following the control phase, a randomized crossover experimental design was employed for the special environment training. The athletes were randomly assigned to one of two groups for Phase 1 of the intervention: one group underwent training under high temperature and high humidity conditions (HOT training, *n* = 4), whereas the other trained under hypoxic conditions (HYP training, *n* = 4). Each environmental training phase lasted for 4 weeks. As in the control phase, body composition assessments and incremental load exercise tests were conducted both pretraining and post‐training.

**FIGURE 1:**
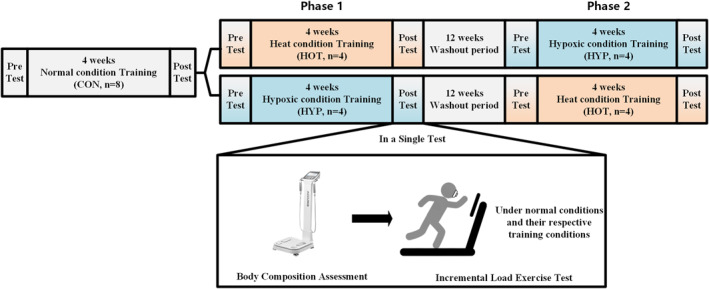
Experimental flowchart.

Upon completion of Phase 1, a 12‐week washout period was implemented to mitigate potential carryover effects between environmental exposures. Subsequently, the athletes proceeded to Phase 2 of the intervention, in which training conditions were crossed over: participants who had previously trained under HOT conditions transitioned to HYP and vice versa. Phase 2 also spanned 4 weeks, and the same preintervention and postintervention assessments were repeated. For analytical purposes, all training data were pooled by the intervention type. Data collected during the HOT training condition were combined across participants, regardless of whether the HOT training occurred in Phase 1 or Phase 2. An identical approach was employed for the HYP training condition. A schematic overview of the experimental protocol is provided in Figure 1.

For the incremental load exercise tests, specific environmental conditions were applied based on the training context:

- For the HOT training phase, tests were conducted under both high temperature and high humidity and normal conditions.
- For the HYP training phase, tests were conducted under both hypoxic and normal conditions.
- For the CON training phase, tests were conducted exclusively under normal conditions.

To control for potential fatigue effects, a 48 h interval was maintained between tests conducted in different environments (Yatsutani et al. 2020).

All tests and training were conducted in low‐altitude regions during spring and autumn to minimize the influence of external environmental factors. Throughout the training period, athletes' daily energy intake was closely monitored to ensure consistency and their diet and hydration were standardized the day before each formal test. Specifically, athletes consumed a predetermined meal plan providing a balanced macronutrient composition (carbohydrates, proteins, and fats) and a controlled water intake based on their body weight and training demands. These measures aimed to reduce potential variability in test outcomes related to pretest nutritional and hydration status.

### 2.3 Environmental Parameters

This study was conducted in the special environment laboratory of the Shanghai Institute of Sports Science. Three distinct exercise environments were established: high temperature and high humidity with normal oxygen (heat condition), normal temperature and low oxygen with normal humidity (hypoxic condition), and a normal environment with normal temperature, humidity, and oxygen (normal condition). The environmental parameters were as follows: Heat condition: ambient temperature of 35°C, relative humidity (RH) of 70%, and oxygen concentration (FiO_2_ = 21.0%). Hypoxic condition: ambient temperature of 23°C, RH of 45%, and FiO_2_ = 15.3%. Normal Condition: ambient temperature of 23°C, RH of 45%, and FiO_2_ = 21.0%.

### 2.4 Training Protocol

Based on the specific demands of modern pentathlon, a training regimen was developed that alternated aerobic endurance running with sprint training, performed 5 times per week in hypoxic condition, heat condition, and normal condition. The aerobic endurance component consisted of three 20 min bouts of treadmill running at moderate intensity (60% VO_2_max), with 10 min rest intervals between sets, for a total of 90 min per session. Sprint training involved high‐intensity sprints (80%–90% VO_2_max) for 1 min, followed by 5 min of moderate‐intensity running (60% VO_2_max), with a 6 min rest between sets. A total of six sets were completed for a session lasting 90 min. During each session, athletes were provided with 1.5 L of water administered in 250 mL doses every 15 min.

### 2.5 Test Protocol

#### 2.5.1 Body Composition Assessment

Athletes' body composition was assessed using a bioimpedance analysis device (InBody 370, Biospace, Korea). Measurements included lean muscle mass, body fat percentage, fat mass, and free fat mass. Prior to testing, athletes were briefed on the procedure. All measurements were conducted by the same trained professional under standardized conditions.

#### 2.5.2 Incremental Load Exercise Protocol

The incremental load exercise test was performed on a treadmill, beginning at a speed of 8 km/h with a 0% incline. Speed was increased by 1 km/h every minute while keeping the incline constant, up to a maximum speed of 18 km/h. Once the speed limit was reached, the incline was increased by 1% per minute. The test was terminated when participants could no longer maintain the required effort (Subudhi et al. 2007). During the test, oxygen uptake (VO_2_), carbon dioxide output (VCO_2_), ventilation (VE), respiratory frequency (RF), tidal volume (VT), and the respiratory exchange ratio (RER) were measured using the COSMED Quark PFT ergo system (OMNIA CPET, Italy). Heart rate (HR) was recorded using the Polar Team PRO heart rate monitor (Polar Electro, Finland).

### 2.6 Data Analysis

VO_2_ and VCO_2_ data from the incremental load exercise tests were smoothed over 10 s intervals. The V‐slope method was used to determine the first ventilatory threshold (VT_1_) (Beaver et al. 1986), and the second ventilatory threshold (VT_2_) was identified based on the inflection point of the VE/VCO_2_ curve (Whipp et al. 1989). The fat oxidation rate at varying exercise intensities, ranging from 30% to 80% VO_2_peak, was calculated at 10% increments (Gagnon et al. 2020).

Fat oxidation rate was computed using data from the gas exchange analyzer and was calculated based on Jeukendrup et al.’s formula (A. Jeukendrup and Wallis 2005) shown in Equation (1). Given that short‐term exercise and environmental stress are unlikely to significantly affect protein metabolism, protein consumption was not considered in this analysis (Vallerand and Jacobs 1989).

(1) $$\text{Fat}\left(g\cdot \min^{-1}\right)=1.695VO_{2}\left(L\cdot \min^{-1}\right)-1.701V{\text{CO}}_{2}\left(L\cdot \min^{-1}\right)$$

MATLAB 2021a (MathWorks, USA) was used to analyze the data. The SIN mathematical model, proposed by Chenevière et al. (2009), was employed to fit the fat oxidation rates across different environments. This model's parameters include dilatation (*d*), symmetry (*s*), and translation (*t*), which describe the shape and shift of the fat oxidation curve along the horizontal axis, as shown in Equation (2).

(2) %MFO=sinπ1sπ+2d·K·%VO2peak+d+ts

where K is the constant π/100 and *d*, *t,* and *s* are the kinetic parameters of fat oxidation, respectively.

### 2.7 Statistical Analysis

Data were analyzed using the SPSS software (version 21.0, IBM Corp., Armonk, NY, USA), with results expressed as mean ± SD. Parametric tests were applied when the data met normal distribution and homogeneity of variance criteria. A two factor repeated measures analysis of variance (ANOVA) was used to evaluate the effects of training (CON, HOT, and HYP) and time points (pretraining and post‐training) on the measured variables. Post hoc comparisons using the Bonferroni correction were conducted to identify pairwise differences between environmental conditions and time points. For abnormally distributed data, nonparametric tests were employed. Statistical significance was set at *α* = 0.05, and a 95% confidence interval was applied.

## 3 Results

### 3.1 Body Composition Test Results

No significant differences in body composition were observed between the trainings (*Ps* > 0.05) as shown in Table 1.

**TABLE 1:** Comparison of body composition of subjects.

Variable	CON		HYP		HOT	
	Pretraining	Post‐training	Pretraining	Post‐training	Pretraining	Post‐training
Weight (kg)	68.84 ± 9.14	68.81 ± 8.82	72.58 ± 8.20	73.24 ± 9.06	72.65 ± 9.39	72.85 ± 7.95
BMI (kg/m^2^)	21.00 ± 1.69	20.97 ± 1.77	22.17 ± 1.81	22.35 ± 2.04	22.22 ± 2.15	22.35 ± 1.81
BF (%)	13.41 ± 2.56	13.04 ± 3.48	13.79 ± 3.77	13.44 ± 3.38	14.48 ± 3.21	13.56 ± 3.25
FM (kg)	13.90 ± 2.80	16.50 ± 4.32	14.03 ± 3.98	13.90 ± 3.65	15.46 ± 3.47	14.12 ± 3.18
FFM (kg)	9.28 ± 2.26	9.16 ± 3.39	10.08 ± 3.13	9.93 ± 2.92	10.61 ± 2.82	10.01 ± 2.71
LMM (kg)	59.56 ± 7.75	59.65 ± 6.08	62.50 ± 7.17	63.31 ± 7.61	62.04 ± 7.73	62.84 ± 6.93

Abbreviations: BF, body fat percentage; BMI, body mass index; FFM, fat‐free mass; FM, fat mass; LMM, lean muscle mass.

### 3.2 Aerobic Metabolic Capacity

#### 3.2.1 Testing in Normal Condition

There was a significant interaction effect of time × training on absolute V̇O_2_ (*F*(2, 21) = 3.754, *p* = 0.040, and *ηp* ^2^ = 0.263), with the HOT training showing an increase of 238.152 mL/min in absolute V̇O_2_ post‐training compared to pretraining (*p* = 0.003 and 95% CI [88.866, 387.438]). A similar interaction effect of time × training was observed for absolute V̇CO_2_ (*F*(2, 21) = 4.325, *p* = 0.027, and *ηp* ^2^ = 0.292), where the HOT training exhibited an increase of 291.183 mL/min in absolute V̇CO_2_ post‐training compared to pretraining (*p* = 0.034 and 95% CI [24.798, 557.568]). Additionally, post‐training absolute V̇CO_2_ in the HOT training was significantly higher by 960.485 mL/min compared to the CON training (*p* = 0.033 and 95% CI [65.972, 1854.997]). For relative V̇CO_2_, there was also a significant interaction effect of time × training (*F*(2, 21) = 4.299, *p* = 0.027, and *ηp* ^2^ = 0.290), with the HOT training showing an increase of 4.051 mL/min/kg in relative V̇CO_2_ post‐training compared to pretraining (*p* = 0.032 and 95% CI [0.393, 7.708]). Furthermore, post‐training relative V̇CO_2_ in the HOT training was significantly higher by 10.156 mL/min/kg compared to the CON training (*p* = 0.021 and 95% CI [1.305, 19.008]).

An interaction effect of time × training was observed for VT_2_@Time (*F*(2, 21) = 3.872, *p* = 0.037, and *ηp* ^2^ = 0.269), where the HOT training showed an increase of 96.062 s in VT_2_@Time post‐training compared to pretraining (*p* = 0.006 and 95% CI [31.471, 160.654]). Similarly, the HYP training exhibited an increase of 109.917 s in VT_2_@Time post‐training compared to pretraining (*p* = 0.002 and 95% CI [45.325, 174.509]). Post‐training, the HOT training had a significant increase of 78.091 s in VT_2_@Time compared to the CON training (*p* = 0.034 and 95% CI [4.794, 151.387]). There was a significant main effect of time on absolute VT_2_ (*F*(1, 21) = 6.701, *p* = 0.017, and *ηp* ^2^ = 0.242), indicating an increase in absolute VT_2_ post‐training compared to pretraining. A similar main effect of time was found for relative VT_2_ (*F*(1, 21) = 4.886, *p* = 0.038, and *ηp* ^2^ = 0.189), showing an increase in relative VT_2_ after the training period.

Lastly, there was a significant interaction effect of time × training on exercise time (*F*(2, 21) = 3.562, *p* = 0.047, and *ηp* ^2^ = 0.253). Post‐training, the HOT training showed a significant increase in exercise time by 46.750 s (*p* = 0.005 and 95% CI [16.178, 77.322]), and the HYP training showed an increase of 58.500 s (*p* = 0.001, 95% CI [27.928 and 89.072]) as shown in Table 2.

**TABLE 2:** Effects of different environment training on aerobic capacity under normal conditions.

Variable	CON		HYP		HOT	
	Pretraining	Post‐training	Pretraining	Post‐training	Pretraining	Post‐training
RF (cpm)	60.15 ± 6.82	57.58 ± 4.54	57.82 ± 12.96	53.91 ± 8.84	58.90 ± 13.17	58.51 ± 9.27
VT (L)	2.55 ± 0.41	2.41 ± 0.34	2.71 ± 0.74	2.97 ± 0.81	2.86 ± 0.98	2.89 ± 0.79
VE (L/min)	149.35 ± 11.54	137.54 ± 18.29	151.16 ± 25.85	156.28 ± 28.84	160.03 ± 28.39	165.56 ± 32.60
IV (L/min)	2427.43 ± 617.94	2508.29 ± 385.09	2588.63 ± 735.88	2836.25 ± 595.85	2645 ± 912.88	2632 ± 723.60
V̇O_2_ (absolute, mL/min)	4104.75 ± 370.48	4072.98 ± 410.84	4402.91 ± 694.36	4447.9 ± 650.06	4322.6 ± 651.64	4560.75 ± 722.64^a^
V̇O_2_ (relative, mL/min/kg)	59.05 ± 4.07	58.91 ± 2.98	60.86 ± 8.37	61.57 ± 7.87	59.40 ± 6.89	62.74 ± 6.84
V̇CO_2_ (absolute, mL/min)	4492.11 ± 353.07	4252.98 ± 474.85	4965.1 ± 745.97	4946.62 ± 683.36	4922.28 ± 815.03	5213.46 ± 852.32^a^ ^,^ ^b^
V̇CO_2_ (relative, mL/min/kg)	64.64 ± 3.68	61.43 ± 2.32	68.74 ± 9.62	68.56 ± 8.97	67.53 ± 8.22	71.59 ± 7.29
RER	1.1 ± 0.03	1.04 ± 0.02	1.13 ± 0.05	1.11 ± 0.05	1.14 ± 0.03	1.14 ± 0.04
VT_1_@time (s)	460.05 ± 111.69	429.89 ± 48.89	425.15 ± 50.61	547.5 ± 83.28	485.13 ± 104.45	523.75 ± 101.27
VT_2_@time (s)	646.6 ± 86.17	644.41 ± 35.36	573.83 ± 51.77	683.75 ± 76.15^a^	626.44 ± 75.58	722.50 ± 49.79^a^ ^,^ ^b^
VT_1_ (absolute, mL/min)	2993.06 ± 358.92	2981.13 ± 546.1	3126.95 ± 316.32	3315.88 ± 450.30	3282.86 ± 333.02	3309.88 ± 439.33
VT_2_ (absolute, mL/min)	3707.93 ± 305.74	3771.13 ± 378.57	3601.32 ± 292.31	3894.63 ± 529.97	3760.33 ± 326.43	4031.13 ± 517.17
VT_1_ (relative, mL/min/kg)	44.22 ± 4.83	43.09 ± 7.07	43.28 ± 4.11	46.00 ± 6.35	45.31 ± 4.48	45.76 ± 5.40
VT_2_ (relative, mL/min/kg)	55.01 ± 1.89	54.73 ± 5.7	50.01 ± 5.49	54.04 ± 7.30	51.88 ± 4.14	55.63 ± 5.37
HR (bpm)	189.63 ± 9.87	185.50 ± 8.23	194.00 ± 6.14	193.00 ± 6.76	192.5 ± 7.67	192.38 ± 9.27
Exercise time (s)	886.63 ± 26.34	892.29 ± 34.55	881.13 ± 41.08	939.63 ± 62.47^a^	894.5 ± 70.57	941.25 ± 33.58^a^

^a^ Statistically significant differences within trainings (*p* < 0.05).

^b^ Statistically significant differences compared with CON (*p* < 0.05).

#### 3.2.2 HOT Training in Heat and Normal Conditions

There was a significant main effect of time on VE (*F*(1, 14) = 8.866, *p* = 0.010, and *ηp* ^2^ = 0.388), with VE increasing post‐training compared to pretraining. A main effect of time was also observed for absolute V̇O_2_ (*F*(1, 14) = 14.708, *p* = 0.002, and *ηp* ^2^ = 0.512), with absolute V̇O_2_ increasing after training compared to before. Similarly, a main effect of time was found for relative V̇O_2_ (*F*(1, 14) = 15.258, *p* = 0.002, and *ηp* ^2^ = 0.521), with relative V̇O_2_ significantly higher post‐training compared to pretraining. For absolute V̇CO_2_, a time × training interaction effect was found (*F*(1, 14) = 5.556, *p* = 0.034, and *ηp* ^2^ = 0.284). Prior to training, absolute V̇CO_2_ was lower in the heat condition compared to the normal condition by 843.122 mL/min (*p* = 0.031, 95% CI [87.799, and 1598.445]). Post‐training, absolute V̇CO_2_ increased by 291.183 mL/min (*p* = 0.041 and 95% CI [14.277, 568.090]) in the normal condition and by 721.542 mL/min (*p* < 0.001 and 95% CI [444.635, 998.449]) in the heat condition. A significant time × training interaction effect was also observed for relative V̇CO_2_ (*F*(1, 14) = 5.026, *p* = 0.042, and *ηp* ^2^ = 0.264). Prior to training, relative V̇CO_2_ was lower in the heat condition by 11.361 mL/min/kg compared to the normal condition (*p* = 0.004 and 95% CI [4.201, 18.521]). After training, relative V̇CO_2_ increased by 10.089 mL/min/kg in the heat condition compared to pretraining (*p* < 0.001 and 95% CI [6.004, 14.174]). There was a main effect of time on RER (*F*(1, 14) = 5.319, *p* = 0.037, and *ηp* ^2^ = 0.275), with RER increasing post‐training compared to pretraining.

For VT_1_@Time, a main effect of time was observed (*F*(1, 14) = 5.432, *p* = 0.035, and *ηp* ^2^ = 0.280), with VT_1_@Time increasing post‐training. A main effect of training was also observed for VT_1_@Time (*F*(1, 14) = 11.130, *p* = 0.005, and *ηp* ^2^ = 0.443), with VT_1_@Time being lower in the heat condition compared to the normal condition. A significant main effect of time was found for VT_2_@Time (*F*(1, 14) = 22.527, *p* < 0.001, and *ηp* ^2^ = 0.617), with VT_2_@Time increasing post‐training compared to pretraining. A main effect of training was also observed for VT_2_@Time (*F*(1, 14) = 15.793, *p* = 0.001, and *ηp* ^2^ = 0.530), with VT_2_@Time being lower in the heat condition compared to the normal condition. For absolute VT_1_, a significant main effect of training was found (*F*(1, 14) = 12.761, *p* = 0.003, and *ηp* ^2^ = 0.477), with absolute VT_1_ being lower in the heat condition compared to the normal condition. A main effect of time was observed for absolute VT_2_ (*F*(1, 14) = 9.390, *p* = 0.008, and *ηp* ^2^ = 0.401), with absolute VT_2_ increasing post‐training compared to pretraining. However, a main effect of training was found for absolute VT_2_ (*F*(1, 14) = 6.708, *p* = 0.021, and *ηp* ^2^ = 0.324), with absolute VT_2_ being lower in the heat condition compared to the normal condition. For relative VT_1_, a significant main effect of training was found (*F*(1, 14) = 17.514, *p* = 0.001, and *ηp* ^2^ = 0.556), with relative VT_1_ being lower in the heat condition compared to the normal condition. There was also a main effect of time for relative VT_2_ (*F*(1, 14) = 10.113, *p* = 0.007, and *ηp* ^2^ = 0.419), with relative VT_2_ increasing post‐training. A significant main effect of training was found for relative VT_2_ (*F*(1, 14) = 11.279, *p* = 0.005, and *ηp* ^2^ = 0.446), with relative VT_2_ being lower in the heat condition compared to the normal condition.

Finally, a main effect of training was observed for exercise time (*F*(1, 14) = 6.451, *p* = 0.024, and *ηp* ^2^ = 0.315), with exercise time being shorter in the heat condition compared to the normal condition, as shown in Table 3.

**TABLE 3:** Effects of high temperature and high humidity environment training on aerobic capacity.

Variable	Heat		Normal	
	Pretraining	Post‐training	Pretraining	Post‐training
RF (cpm)	54.88 ± 7.02	59.32 ± 11.20	58.90 ± 13.17	58.51 ± 9.27
VT (L)	2.74 ± 0.74	2.85 ± 0.75	2.86 ± 0.98	2.89 ± 0.79
VE (L/min)	148.20 ± 31.31	163.8 ± 25.56	160.03 ± 28.39	165.56 ± 32.60
IV (L/min)	2626.88 ± 541.82	2673.75 ± 554.28	2645 ± 912.88	2632 ± 723.60
V̇O_2_ (absolute, mL/min)	3824.74 ± 730.97	4174.47 ± 641.35	4322.6 ± 651.64	4560.75 ± 722.64
V̇O_2_ (relative, mL/min/kg)	52.75 ± 8.47	57.58 ± 7.78	59.40 ± 6.89	62.74 ± 6.84
V̇CO_2_ (absolute, mL/min)	4079.16 ± 572.63^b^	4800.70 ± 541.30^a^	4922.28 ± 815.03	5213.46 ± 852.32^a^
V̇CO_2_ (relative, mL/min/kg)	56.17 ± 4.65^b^	66.26 ± 6.21^a^	67.53 ± 8.22	71.59 ± 7.29
RER	1.08 ± 0.10	1.16 ± 0.07	1.14 ± 0.03	1.14 ± 0.04
VT_1_@time (s)	339.02 ± 74.66	441.25 ± 81.32	485.13 ± 104.45	523.75 ± 101.27
VT_2_@time (s)	512.69 ± 63.06	635.00 ± 80.89	626.44 ± 75.58	722.50 ± 49.79
VT_1_ (absolute, mL/min)	2518.2 ± 458.13	2849.13 ± 346.92	3282.86 ± 333.02	3309.88 ± 439.33
VT_2_ (absolute, mL/min)	3122.3 ± 483.64	3567.5 ± 578.09	3760.33 ± 326.43	4031.13 ± 517.17
VT_1_ (relative, mL/min/kg)	35.02 ± 5.59	39.30 ± 3.50	45.31 ± 4.48	45.76 ± 5.40
VT_2_ (relative, mL/min/kg)	43.51 ± 6.37	49.03 ± 5.19	51.88 ± 4.14	55.63 ± 5.37
HR (bpm)	189.63 ± 13.04	193.88 ± 6.08	192.5 ± 7.67	192.38 ± 9.27
Exercise time (s)	808.88 ± 127.55	856.38 ± 82.18	894.50 ± 70.57	941.25 ± 33.58

*Note:* “Heat” and “Normal” indicate measurements conducted under heat and normal conditions, respectively, before and after HOT training.

^a^ Statistically significant differences within trainings (*p* < 0.05).

^b^ Statistically significant differences compared with normal conditions (*p* < 0.05).

#### 3.2.3 HYP Training in Hypoxic and Normal Conditions

A significant main effect of training was found for relative V̇O_2_ (*F*(1, 14) = 6.350, *p* = 0.025, and *ηp* ^2^ = 0.312), with relative V̇O_2_ being lower in the hypoxic condition compared to the normal condition. Similarly, a main effect of training was observed for relative V̇CO_2_ (*F*(1, 14) = 5.409, *p* = 0.036, and *ηp* ^2^ = 0.279), with relative V̇CO_2_ being lower in the hypoxic condition compared to the normal condition.

For VT_1_@Time, there was a significant interaction effect of time × training (*F*(1, 14) = 6.020, *p* = 0.028, and *ηp* ^2^ = 0.301). Post‐training, VT_1_@Time increased by 122.354 s in the normal condition compared to pretraining (*p* = 0.002 and 95% CI [53.550, 191.159]). However, in the hypoxic condition, VT_1_@Time decreased by 153.750 s post‐training compared to pretraining (*p* = 0.002 and 95% CI [67.131, 240.369]). A main effect of time was found for VT_2_@Time (*F*(1, 14) = 27.089, *p* < 0.001, and *ηp* ^2^ = 0.659), with VT_2_@Time increasing post‐training compared to pretraining. Additionally, there was a main effect of training for VT_2_@Time (*F*(1, 14) = 7.865, *p* = 0.014, and *ηp* ^2^ = 0.360), with VT_2_@Time being lower in the hypoxic condition compared to the normal condition. A significant main effect of time was observed for absolute VT_2_ (*F*(1, 14) = 7.833, *p* = 0.014, and *ηp* ^2^ = 0.359), with absolute VT_2_ increasing post‐training compared to pretraining. Similarly, a main effect of time was found for relative VT_2_ (*F*(1, 14) = 6.941, *p* = 0.020, and *ηp* ^2^ = 0.331), with relative VT_2_ increasing post‐training compared to pretraining.

There was a significant main effect of training on HR (*F*(1, 14) = 6.798, *p* = 0.021, and *ηp* ^2^ = 0.327), with HR being lower in the hypoxic condition compared to the normal condition. For exercise time, a significant main effect of time was observed (*F*(1, 14) = 22.736, *p* < 0.001, and *ηp* ^2^ = 0.619), with exercise time increasing post‐training compared to pretraining. Additionally, there was a significant main effect of training on exercise time (*F*(1, 14) = 11.188, *p* = 0.005, and *ηp* ^2^ = 0.444), with exercise time being shorter in the hypoxic condition compared to the normal condition, as shown in Table 4.

**TABLE 4:** Effects of hypoxic environment training on aerobic capacity.

Variable	Hypoxia		Normal	
	Pretraining	Post‐training	Pretraining	Post‐training
RF (cpm)	58.31 ± 7.51	55.84 ± 8.41	57.82 ± 12.96	53.91 ± 8.84
VT (L)	2.64 ± 0.43	2.83 ± 0.64	2.71 ± 0.74	2.97 ± 0.81
VE (L/min)	153.12 ± 24.87	155.41 ± 24.83	151.16 ± 25.85	156.28 ± 28.84
IV (L/min)	2726.38 ± 441.56	2725.63 ± 672.70	2588.63 ± 735.88	2836.25 ± 595.85
V̇O_2_ (absolute, mL/min)	3741.06 ± 388.12	3958.84 ± 585.56	4402.91 ± 694.36	4447.9 ± 650.06
V̇O_2_ (relative, mL/min/kg)	51.79 ± 3.81	54.25 ± 6.51	60.86 ± 8.37	61.57 ± 7.87
V̇CO_2_ (absolute, mL/min)	4298.41 ± 588.88	4526.49 ± 641.85	4965.1 ± 745.97	4946.62 ± 683.36
V̇CO_2_ (relative, mL/min/kg)	59.23 ± 3.76	62.05 ± 7.19	68.74 ± 9.62	68.56 ± 8.97
RER	1.14 ± 0.05	1.14 ± 0.02	1.13 ± 0.05	1.10 ± 0.06
VT_1_@time (s)	382.71 ± 69.46	393.75 ± 78.18^b^	425.15 ± 50.61^a^	547.5 ± 83.28
VT_2_@time (s)	507.67 ± 89.07	563.75 ± 72.69	573.83 ± 51.77	683.75 ± 76.15
VT_1_ (absolute, mL/min)	2922.78 ± 316.22	2888.13 ± 433.16	3126.95 ± 316.32	3315.88 ± 450.3
VT_2_ (absolute, mL/min)	3247.37 ± 387	3473.75 ± 549.60	3601.32 ± 292.31	3894.63 ± 529.97
VT_1_ (relative, mL/min/kg)	40.54 ± 4.05	40.03 ± 5.45	43.28 ± 4.11	46.00 ± 6.35
VT_2_ (relative, mL/min/kg)	45.05 ± 4.78	48.15 ± 7.03	50.01 ± 5.49	54.04 ± 7.30
HR (bpm)	188.13 ± 6.31	184.25 ± 5.65	194.00 ± 6.14	193.00 ± 6.76
Exercise time (s)	769.63 ± 47.11	883.25 ± 86.48	881.13 ± 41.08	939.63 ± 62.47

*Note:* “Hypoxia” and “Normal” indicate measurements conducted under hypoxic and normal conditions, respectively, before and after HYP training.

^a^ Statistically significant differences within trainings (*p* < 0.05).

^b^ Statistically significant differences compared with normal conditions (*p* < 0.05).

### 3.3 Kinetic Parameters of Fat Oxidation

**FIGURE 2:**
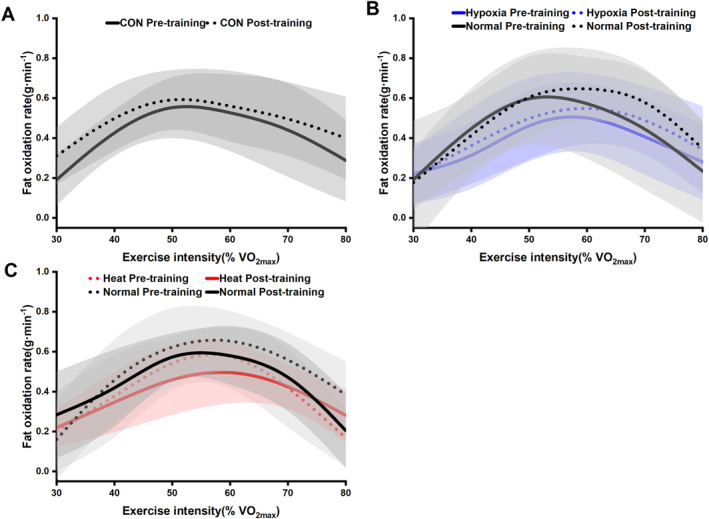
Effects of different environmental trainings on fat oxidation curves. Fat oxidation curves before and after training for the CON group. The solid black line represents the fat oxidation curve under Normal conditions before training, while the dashed black line shows the curve after training (A). Fat oxidation curves before and after training for the HYP group. The solid black line represents the fat oxidation curve under Normal conditions before training, while the dashed black line shows the curve after training. The solid blue line represents the fat oxidation curve under Hypoxic conditions before training, and the dashed blue line shows the curve after training (B). Fat oxidation curves before and after training for the HOT group. The solid black line represents the fat oxidation curve under Normal conditions before training, while the dashed black line shows the curve after training. The solid red line represents the fat oxidation curve under Heat conditions before training, and the dashed red line shows the curve after training (C).

We assessed the changes in the fat oxidation rate curves of athletes after training in different environments. We found that, compared to pretraining values, the HOT training exhibited an upward shift and slight expansion of the fat oxidation curve under heat conditions as well as under normal conditions post‐training. Similarly, the HYP training showed an upward shift and slight expansion of the fat oxidation curve under normal conditions after training compared to pretraining. In subsequent analyses, we used the SIN mathematical model to fit the fat oxidation curves and further analyzed the characteristic parameters of these curves as shown in Figure 2.

#### 3.3.1 Testing in Normal Condition

There was a significant time × training interaction effect for MFO (*F*(2, 21) = 7.179, *p* = 0.004, and *ηp* ^2^ = 0.406). Post‐training, MFO increased by 0.126 g/min in the HOT training compared to pretraining (*p* = 0.015 and 95% CI [0.027, 0.226]) and by 0.157 g/min in the HYP training (*p* = 0.004 and 95% CI [0.057, 0.256]). A main effect of time was found for MFO @ V̇O_2_ (*F*(1, 21) = 8.146, *p* = 0.009, and *ηp* ^2^ = 0.279), with MFO @ V̇O_2_ increasing post‐training compared to pretraining. There was a significant time × training interaction effect for FAT_max_ (*F*(2, 21) = 4.484, *p* = 0.024, and *ηp* ^2^ = 0.299). Post‐training, FAT_max_ increased by 5.303 g/min in the HOT training compared to pretraining (*p* = 0.005 and 95% CI [1.753, 8.852]). For *dilatation*, a time × training interaction effect was observed (*F*(2, 21) = 3.714, *p* = 0.042, and *ηp* ^2^ = 0.261). Post‐training, *dilatation* increased by 0.290 in the HOT training (*p* = 0.003 and 95% CI [0.113, 0.467]) and by 0.283 in the HYP training (*p* = 0.003 and 95% CI [0.106, 0.459]) compared to pretraining. A main effect of time was observed for *translation* (*F*(1, 21) = 5.995, *p* = 0.023, and *ηp* ^2^ = 0.222), with *translation* decreasing post‐training compared to pretraining, as shown in Table 5.

**TABLE 5:** Effects of different environmental training on fat oxidation under normal conditions.

Variable	CON		HYP		HOT	
	Pretraining	Post‐training	Pretraining	Post‐training	Pretraining	Post‐training
MFO (g/min)	0.67 ± 0.23	0.59 ± 0.14	0.63 ± 0.17	0.79 ± 0.18^a^	0.65 ± 0.14	0.78 ± 0.13 ^a^
MFO@ V̇O_2_ (%)	53.49 ± 6.59	54.07 ± 6.89	51.09 ± 5.74	55.85 ± 6.12	53.10 ± 6.02	59.32 ± 3.23
FAT_max_ (mL/min·kg)	33.54 ± 1.56	31.77 ± 4.54	31.37 ± 2.63	34.43 ± 3.97	30.46 ± 3.61	35.76 ± 5.12^a^
Symmetry	0.77 ± 0.17	0.93 ± 0.22	0.73 ± 0.16	0.82 ± 0.11	0.85 ± 0.11	0.88 ± 0.26
Dilatation	−0.55 ± 0.13	−0.55 ± 0.10	−0.79 ± 0.27	−0.51 ± 0.12^a^	−0.74 ± 0.28	−0.45 ± 0.16^a^
Translation	−0.06 ± 0.25	−0.13 ± 0.13	−0.04 ± 0.29	−0.32 ± 0.23	−0.11 ± 0.52	−0.35 ± 0.16

Abbreviations: %MFO, relative oxygen uptake at MFO (% of V̇O_2_peak); FAT_max_, exercise intensity at MFO (mL/min·kg); MFO, maximum fat oxidation rate (g/min).

^a^ Statistically significant differences within trainings (*p* < 0.05).

#### 3.3.2 HOT Training in Heat and Normal Conditions

A significant main effect of time was observed for MFO (*F*(1, 14) = 27.122, *p* < 0.001, and *ηp* ^2^ = 0.660), with MFO increasing post‐training compared to pretraining. There was also a main effect of time for MFO @ V̇O_2_ (*F*(1, 14) = 6.683, *p* = 0.022, and *ηp* ^2^ = 0.323), with MFO @ V̇O_2_ increasing post‐training compared to pretraining. For FAT_max_, a significant main effect of time was found (*F*(1, 14) = 7.701, *p* = 0.015, and *ηp* ^2^ = 0.355), with FAT_max_ increasing post‐training compared to pretraining. A main effect of time was observed for *dilatation* (*F*(1, 14) = 8.050, *p* = 0.013, *ηp* ^2^ = 0.365), with *dilatation* increasing post‐training compared to pretraining, as shown in Table 6.

**TABLE 6:** Influence of high temperature and humidity environment training on fat oxidation.

Variable	Heat		Normal	
	Pretraining	Post‐training	Pretraining	Post‐training
MFO (g/min)	0.56 ± 0.17	0.65 ± 0.09	0.65 ± 0.14	0.78 ± 0.13
MFO@ V̇O_2_ (%)	54.22 ± 3.33	55.75 ± 6.30	53.1 ± 6.02	59.32 ± 3.23
FAT_max_ (mL/min·kg)	28.81 ± 5.28	32.55 ± 7.45	30.46 ± 3.61	35.76 ± 5.12
Symmetry	0.83 ± 0.14	0.96 ± 0.16	0.85 ± 0.11	0.88 ± 0.26
Dilatation	−0.40 ± 0.15	−0.34 ± 0.12	−0.74 ± 0.28	−0.45 ± 0.16
Translation	−0.40 ± 0.09	−0.29 ± 0.21	−0.11 ± 0.52	−0.35 ± 0.16

*Note:* “Heat” and “Normal” indicate measurements conducted under heat and normal conditions, respectively, before and after HOT training.

Abbreviations: %MFO, relative oxygen uptake at MFO (% of V̇O_2_peak); FAT_max_, exercise intensity at MFO (mL/min·kg); MFO, maximum fat oxidation rate (g/min).

#### 3.3.3 HYP Training in Hypoxic and Normal Conditions

A significant time × training interaction effect was found for MFO (*F*(1, 14) = 7.701, *p* = 0.015, and *ηp* ^2^ = 0.355). Post‐training, MFO increased by 0.156 g/min under normal conditions compared to pretraining (*p* < 0.001 and 95% CI [0.091, 0.221]). For MFO @ V̇O_2_, a time × training interaction effect was observed (*F*(1, 14) = 4.758, *p* = 0.047, and *ηp* ^2^ = 0.254). Post‐training, MFO @ V̇O_2_ increased by 4.764% under normal conditions compared to pretraining (*p* = 0.010 and 95% CI [1.354, 8.173]). A main effect of time was observed for FAT_max_ (*F*(1, 14) = 8.434, *p* = 0.012, and *ηp* ^2^ = 0.376), with FAT_max_ increasing post‐training compared to pretraining. For *dilatation*, a time × training interaction effect was found (*F*(1, 14) = 4.909, *p* = 0.044, and *ηp* ^2^ = 0.260). Post‐training, *dilatation* increased by 0.283 under normal conditions compared to pretraining (*p* = 0.008 and 95% CI [0.087, 0.478]) as shown in Table 7.

**TABLE 7:** Effects of hypoxic environment training on fat oxidation.

Variable	Hypoxia		Normal	
	Pretraining	Post‐training	Pretraining	Post‐training
MFO (g/min)	0.56 ± 0.17	0.60 ± 0.17	0.63 ± 0.17	0.79 ± 0.18^a^
MFO@ V̇O_2_ (%)	56.95 ± 7.05	56.81 ± 7.33	51.09 ± 5.74	55.85 ± 6.12^a^
FAT_max_ (mL/min·kg)	30.05 ± 4.34	31.01 ± 4.36	31.37 ± 2.63	34.43 ± 3.97
Symmetry	1.06 ± 0.46	0.84 ± 0.22	0.73 ± 0.16	0.82 ± 0.11
Dilatation	−0.62 ± 0.16	−0.62 ± 0.18	−0.79 ± 0.27	−0.51 ± 0.12^a^
Translation	−0.06 ± 0.44	−0.28 ± 0.21	−0.04 ± 0.29	−0.32 ± 0.23

*Note:* “Heat” and “Normal” indicate measurements conducted under heat and normal conditions, respectively, before and after HOT training.

Abbreviations: %MFO, relative oxygen uptake at MFO (% of V̇O_2_peak); FAT_max_, exercise intensity at MFO (mL/min·kg); MFO, maximum fat oxidation rate (g/min).

^a^ Statistically significant differences within trainings (*p* < 0.05).

## 4 Discussion

Our findings demonstrate that a four‐week training program conducted under combined heat and hypoxic conditions significantly enhances athletes' aerobic endurance. Moreover, regarding fat metabolism, we observed a marked increase in MFO and a rightward shift in the fat oxidation curve during exercise under normal conditions following the environmental stress training. These results support our initial hypothesis, suggesting that training under environmental stressors can promote metabolic adaptations conducive to improved athletic performance. However, variations in the specific training environments yielded differential impacts on aerobic metabolism and fat oxidation capacity in both normal and corresponding environments. Notably, training in heat conditions may exert a more pronounced beneficial effect on aerobic metabolism and fat oxidation capacity compared to hypoxia training.

Our results indicate that both four‐week training regimens in heat as well as in hypoxic conditions improved exercise time to exhaustion during normal incremental load tests. We specifically observed an increase in maximal oxygen uptake in both normal and high temperature and high humidity environments following training. Conversely, the hypoxic training did not yield significant enhancements in oxygen uptake, aligning with previous research on the effects of high temperature, high humidity, and hypoxic conditions (Diebel et al. 2017; Henderson et al. 2001; Kettunen et al. 2023). Training in high temperature and humidity conditions facilitates numerous positive physiological adaptations, particularly related to thermoregulation and cardiovascular responses, ultimately aimed at enhancing heat dissipation. Post‐training, athletes frequently exhibit earlier dilation of skin blood vessels, increased sweating rates, and enhanced plasma volume (Magalhães et al. 2010; Meng et al. 2019). These adaptations contribute to a reduction in core body temperature during exercise, heightened sweating sensitivity, and improved cardiovascular stability, which collectively enhance oxygen transport capacity and elevate both endurance levels and V̇O_2_max under normal and high temperature and high humidity conditions (Diebel et al. 2017). Conversely, extensive literature on altitude and hypoxia training suggests that adaptation to hypoxic stimuli can also enhance exercise performance (Geiser et al. 2001; Levine and Stray‐Gundersen 1992). Hypoxic training promotes acute arterial vasodilation and increases blood flow within the skeletal muscle vascular bed by enhancing cardiac output (Jung et al. 2020), increasing hemoglobin mass (Kettunen et al. 2023), and activating nitric oxide (NO) mediated mechanisms during exercise (Vedam et al. 2009). These physiological changes can bolster athletes' endurance performance in both normal and hypoxic conditions. However, the specific alterations in tissue‐level oxygen transport resulting from hypoxic stimuli may not be adequately reflected in V̇O_2_max levels, which could hinder the short‐term enhancement of V̇O_2_max (Ingjer and Myhre 1992; Schmutz et al. 2010).

It is noteworthy that both the HOT and HYP trainings exhibited a delay in VT_2_@Time during tests in normal condition, along with an elevation in ventilatory anaerobic threshold (VAT) in both normal and corresponding environmental conditions, which is consistent with previous findings (Puype et al. 2013; Tyler et al. 2016). Tyler et al. (2016) suggested that training in a heat condition can reduce oxygen consumption during exercise, enhance power output at the lactate threshold, and decrease lactic acid concentration, thereby improving aerobic metabolic efficiency. Furthermore, training in hypoxic conditions has been shown to upregulate muscle phosphofructokinase activity, elevate the anaerobic threshold, and enhance lactic acid metabolism (Oriishi et al. 2018; Puype et al. 2013). The observed delay in the anaerobic ventilatory threshold may be associated with a greater reliance on aerobic energy supply during exercise. By analyzing the fat oxidation curve during incremental exercise tests, we found that fat oxidation capacity increased after training in both heat as well as in hypoxic conditions. This suggests an expansion of the fat oxidation curve and an extension of the duration of fat utilization for energy, thereby improving athletes' endurance. Previous studies have established that the body's fat oxidation capacity is influenced by factors such as blood flow in adipose tissue, lactate threshold, and blood lactate concentration. A decrease in FFA concentration is often attributed to reduced blood flow to adipose tissue, which can limit fat oxidation capacity. Additionally, an increase in anaerobic glycolytic energy supply can disrupt skeletal muscle homeostasis, further impairing fat oxidation (Achten and Jeukendrup 2003). During anaerobic glycolysis, the increased release of hydrogen ions (H^+^) into the bloodstream reduces muscle pH, inhibiting the activity of carnitine palmitoyl transferase I, which further limits fat oxidation (A. E. Jeukendrup 2002; Starritt et al. 2000). Moreover, Périard et al. (2015) found that during submaximal exercise, a decrease in blood and muscle lactate accumulation, along with an increase in power output at the lactate threshold, was associated with reduced muscle glycogen utilization and enhanced fat oxidation. Although our results indicate that the HOT training experienced delayed anaerobic ventilatory thresholds and expanded fat oxidation curves even in heat conditions, the HYP training did not exhibit similar metabolic improvements in the hypoxic condition. This may be due to insufficient enhancements in oxygen transport and utilization under hypoxic conditions. Furthermore, oxygen transport and mitochondrial utilization in body tissues may be directly related to fat oxidation capacity (Zurbuchen et al. 2020). Exposure to hypoxic conditions may further restrict aerobic energy supply and increase the conversion of pyruvate to lactic acid (Wadley et al. 2006).

By comparing MFO, FAT_max_, and other parameters, we observed that training in high temperature, high humidity, and hypoxic conditions significantly increased MFO in both the HOT and HYP trainings. Notably, the increase in FAT_max_ in the HOT training suggests a beneficial enhancement in fat oxidation capacity during tests in normal conditions, consistent with previous findings (Febbraio et al. 1994; Young et al. 1982; Zhang et al. 2023). The current literature supports the idea that metabolic adaptations to training in heat conditions show relative consistency. For example, a study on overweight individuals demonstrated that 10 consecutive days of heat exposure effectively reduced fasting blood glucose and insulin levels. This response triggers greater insulin‐mediated inhibition of hepatic glucose production and nonesterified FFA levels, thereby enhancing fat oxidation (Pallubinsky et al. 2020). Foundational research has also shown that adaptation to high temperatures increases the oxidation rates of fatty acids and glycerol‐3‐phosphate in skeletal muscle mitochondria in rats (Zoladz et al. 2017). Furthermore, increased FFA oxidation may inhibit muscle glycogen utilization and delay the onset of exercise‐induced fatigue (Costill et al. 1977). In contrast, the effects of hypoxic training on fat oxidation capacity remain debated. Vedam et al. (Tonini et al. 2011) reported that after 14 nights of hypoxic exposure, MFO@V̇O_2_ increased during incremental exercise tests, whereas blood lactate concentrations decreased at equivalent exercise intensities. This could be attributed to increases in hematocrit and hemoglobin mass following hypoxic exposure, which enhance tissue oxygenation, thereby reducing blood lactate levels and increasing MFO@V̇O_2_ (Kettunen et al. 2023). A study investigating the effects of high‐altitude exposure on energy metabolism during exercise also found a decrease in muscle glycogen utilization and an increase in free fatty acid utilization postexposure (Young et al. 1982). However, other studies offer contrasting findings. Roels et al. (2007) reported alterations in skeletal muscle mitochondrial function related to substrate oxidation after three weeks of training in a hypoxic environment, with increased glycogen uptake and reduced reliance on fat as an energy source. These discrepancies may be due to variations in the selection of fatty acid metabolic enzymes between different study, such as the content or activity of *β*‐hydroxyacyl‐CoA dehydrogenase or carnitine palmitoyl transferase‐1 (CPT‐1), which may not exhibit significant changes following adaptation to hypoxic conditions (Horscroft and Murray 2014). Notably, the content of carnitine palmitoyl transferase‐2 (CPT‐2) increased relative to citrate synthase and total peptide levels after hypoxic adaptation, indicating a specific enhancement in the body's capacity to oxidize long‐chain acyl‐CoA derivatives. This adaptation enables cells to utilize fatty acids more efficiently as energy sources, thereby improving overall fat oxidation capacity (Chicco et al. 2018).

In our comparison of fat oxidation capacity during exercise under special environmental conditions, we found that the HOT training showed consistent increases in MFO, FAT_max_, and MFO@V̇O_2_ under normal and heat conditions. In contrast, the HYP training demonstrated an increase in FAT_max_ under normal and hypoxic condition, but training did not result in improvements in MFO or MFO@V̇O_2_. Previous studies have shown that acute exercise in both hypoxic conditions (Murray 2016; Workman and Basset 2012) and heat conditions (Ruíz‐Moreno et al. 2021) reduces the body's reliance on fat oxidation. Our findings suggest that long‐term training in heat conditions can mitigate the negative effects of these environmental factors on energy metabolism, which is consistent with the results of King et al. (1985) and Febbraio et al. (1994). However, in the HYP training, only FAT_max_ increased under normal and hypoxic conditions following training. This may be attributed to the inability to offset the negative effects of hypoxic exposure. We hypothesize that the physiological responses to hypoxia differ fundamentally from those triggered by heat, particularly in terms of the gradual core temperature adjustments seen in the latter environment (Nybo et al. 2001). In contrast, exposure to hypoxia exacerbates the imbalance in oxygen transport and utilization during exercise. Additionally, hypoxia training did not lead to sufficient improvements in aerobic metabolic capacity, which likely explains the lack of enhancement in fat oxidation ability during testing in hypoxic conditions in this study. Although we observed enhancements in the athletes' metabolic characteristics during exercise, no corresponding changes in body composition were detected. Despite controlling the athletes' diets throughout the training period, it is important to note that the participants were professional athletes and the special environmental training accounted for a relatively small portion of their overall daily training regimen. As a result, this limited exposure may not have been sufficient to produce measurable changes in body composition.

Our study has several limitations that should be acknowledged. First of all, we acknowledge that using bioelectrical impedance analysis (BIA) for body composition assessment may introduce bias into the results due to its sensitivity to factors such as hydration status, recent physical activity, and environmental conditions. However, BIA remains a practical, noninvasive, and widely used method in field‐based settings, offering reasonable accuracy for training‐level comparisons when standardized protocols are followed (Feng et al. 2024). Secondly, in research on fat oxidation kinetics, a 3 min incremental load exercise protocol is commonly employed to achieve metabolic homeostasis (Chenevière et al. 2010; Gagnon et al. 2020). However, some studies suggest that a 1 min incremental load protocol can also effectively capture FAT_max_ and MFO (Purge et al. 2014). In this study, we chose the 1 min interval protocol, considering the specific characteristics of the sporting events and the athletes' performance levels, to better assess the effects of environmental factors on fat oxidation. Additionally, our investigation focused on specific environmental parameters, such as temperature, humidity, and oxygen concentration, without exploring the impact of variations within these conditions. Consequently, the effects of training under different conditions within heat conditions, as well as hypoxic conditions, on athletes' energy metabolism remain unclear. Future research should incorporate a wider range of environmental variables and exercise protocols to offer a more comprehensive understanding of their influence on athletes' metabolic responses.

## 5 Conclusion

Both 4 weeks of training in a hypoxic environment and in a high temperature and high humidity environment enhance athletes' aerobic metabolic capacity, resulting in improved fat oxidation during tests conducted in normal environmental conditions. Moreover, training in high temperature and high humidity environments offers similar advantages for performance in corresponding specific conditions. These adaptations are particularly beneficial for endurance athletes, as they help delay the onset of exercise‐induced fatigue, enhance aerobic energy production, and extend exercise duration. However, training in a hypoxic environment did not result in improved fat oxidation capacity under hypoxic conditions. Therefore, it is crucial for coaches, medical professionals, and sports scientists to develop specialized nutritional strategies tailored to meet the specific energy metabolism needs of competitive athletes in different environmental settings.

## Ethics Statement

This study was performed in line with the principles of the Declaration of Helsinki. Approval was granted by the Scientific Research Ethics Committee of the Shanghai Institute of Sports Science (Shanghai Anti‐Doping Center) (approval number: LLSC20220005).

## Consent

Informed consent was obtained from all individual participants included in the study.

## Conflicts of Interest

The authors declare no conflicts of interest.

## Data Availability

The datasets generated during and analyzed during the current study are available from the corresponding author on reasonable request.
